# Investigating the Mediating Role of Paranoid Delusions in Depression and Cognitive Decline

**DOI:** 10.1002/alz70860_098151

**Published:** 2025-12-23

**Authors:** Saurabh Kalra, Deepak Kalra, Hannah Gardener, Carolina Marinovic Gutierrez, Mohammad Nafeli Shahrestani, Debina Laishram, Karlon Howard Johnson, Nandakumar Nagaraja, WayWay M. Hlaing, Tatjana Rundek

**Affiliations:** ^1^ University of Miami Miller School of Medicine, Miami, FL, USA; ^2^ Pennsylvania State University College of Medicine, Hershey, PA, USA; ^3^ Evelyn F. McKnight Brain Institute, Miami, FL, USA

## Abstract

**Background:**

Psychotic symptoms, including delusions, are prevalent in Alzheimer's disease and depression, where they are associated with poorer outcomes. Cognitive Load Theory posits that depression impairs cognitive function by increasing rumination and negative thought patterns, depleting cognitive resources. The role of delusions as a mediator between depression and cognitive outcomes remains unexplored.

**Methods:**

Using data from the National Alzheimer's Coordinating Center (NACC), we examined the mediating role of paranoid delusions in the relationship between depression severity and cognitive outcomes. Data were collected from 14,588 participants during their first visit to an Alzheimer's Disease Center (March 2015‐Dec 2024, NACC Version 3). Cognitive function was evaluated using the Montreal Cognitive Assessment (MoCA). Depression severity was proxy‐reported by caregivers using the Neuropsychiatric Inventory Questionnaire (NPI‐Q), while paranoid delusion severity was self‐reported based on beliefs about others stealing or planning harm. Structural equation modeling (SEM) was employed to estimate direct, indirect, and total effects while adjusting for covariates informed by a theoretical framework. These covariates included sex, age, race, education, body mass index, living arrangement, independence, residence type, alcohol and tobacco use, diabetes, heart failure, hypertension, and stroke. Model fit was assessed using root mean square error, comparative fit index, and standardized residuals.

**Results:**

Participants had a mean age of 69.3 years, with 57.4% female, 78.5% White, 17.5% Black or African American, and 4.0% from other racial groups. The average MoCA score was 23 (median = 24, SD=5.8). Overall, 26% had mild or greater depressive symptoms, and 4.6% exhibited mild or greater delusions. Delusion prevalence increased with depression severity: 1.3% in those without depressive symptoms, 7.7% in mild, 14.5% in moderate, and 28% in severe depression. Depression severity was significantly associated with delusions (β=0.092, *p* <0.001), poor cognitive outcomes (β=−0.79, *p* <0.001), and indirectly via delusional severity (β_indirect=−0.188, *p* <0.001). Delusions mediated 19.3% of the total effect of depression severity on cognitive outcomes. The SEM model demonstrated an excellent fit.

**Conclusions:**

The poorer cognitive outcomes in patients with depression are partially mediated through delusions. Given the observational design, this needs to be confirmed through prospective studies and may provide an avenue to improve cognitive outcomes in patients with depression.

